# Static friction coefficient depends on the external pressure and block shape due to precursor slip

**DOI:** 10.1038/s41598-023-29764-w

**Published:** 2023-02-13

**Authors:** Wataru Iwashita, Hiroshi Matsukawa, Michio Otsuki

**Affiliations:** 1 Department of Mechanical Science and Bioengineering, Osaka University, Toyonaka, 560-8531 Japan; 2 Department of Physical Sciences, Aoyama Gakuin University, Sagamihara, 252-5258 Japan

**Keywords:** Condensed-matter physics, Statistical physics, thermodynamics and nonlinear dynamics, Mechanical properties, Mechanical engineering

## Abstract

Amontons’ law states that the maximum static friction force on a solid object is proportional to the loading force and is independent of the apparent contact area. This law indicates that the static friction coefficient does not depend on the external pressure or object shape. Here, we numerically investigate the sliding motion of a 3D viscoelastic block on a rigid substrate using the finite element method (FEM). The macroscopic static friction coefficient decreases with an increase in the external pressure, length, or width of the object, which contradicts Amontons’ law. Precursor slip occurs in the 2D interface between the block and substrate before bulk sliding. The decrease in the macroscopic static friction coefficient is scaled by the critical area of the precursor slip. A theoretical analysis of the simplified models reveals that bulk sliding results from the instability of the quasi-static precursor slip caused by velocity-weakening local friction. We also show that the critical slip area determines the macroscopic static friction coefficient, which explains the results of the FEM simulation.

## Introduction

A friction force prevents the relative sliding motion between two objects in contact. Friction plays a crucial role in various situations, such as the contact surface between the ground and the sole of a shoe, brakes and bearings in machines, and tectonic plates that cause earthquakes. Many studies on friction have been conducted, but the elucidation of the fundamental mechanism of friction is essential for science and technology^1–7^.

Amontons’ law states that the maximum static friction force on a solid object is independent of the apparent contact area and proportional to the load^1–7^. This law has been taught in high school physics textbooks and is believed to hold true for diverse systems. When the friction force obeys Amontons’ law, the friction coefficient, which is the ratio of the friction force to the loading force, does not depend on the pressure, size, or object shape. On a rough frictional interface with numerous asperities, only a tiny fraction of the surfaces forms junctions, the so-called real contact points. Amontons’ law is explained by the proportionality of the total area of real contact points to the loading force^1–9^.

The above explanation for the origin of Amontons’ law implicitly assumes uniformity of the stress field. Therefore, Amontons’ law is not expected to hold if a macroscopic deformation exists. In fact, recent numerical studies have reported the breakdown of Amontons’ law in macroscopic viscoelastic objects^10,11^, revealing that it is related to local quasi-static precursor slips before the onset of bulk sliding owing to non-uniform deformation^10–28^. The relationship between precursor slips and the breakdown of Amontons’ law has been confirmed previously in an experiment with an acrylic glass block^12^. However, previous studies have only investigated systems with a 1D frictional interface. Friction usually occurs in 2D interfaces of 3D objects. However, it is not clear whether the results in previous studies apply to more realistic 3D systems.

In this study, we numerically investigate the sliding motion of a 3D viscoelastic object on a rigid substrate using the finite element method (FEM). The macroscopic static friction coefficient decreases with an increase in the pressure or size of the object. The precursor slip propagates in a 2D frictional interface. Bulk sliding occurs when the area of the precursor slip reaches a critical value, which determines the macroscopic static friction coefficient. An analysis of the simplified models reveals that the instability of the precursor slip leads to bulk sliding.

## Results

### 3D FEM simulation

**Figure 1:**
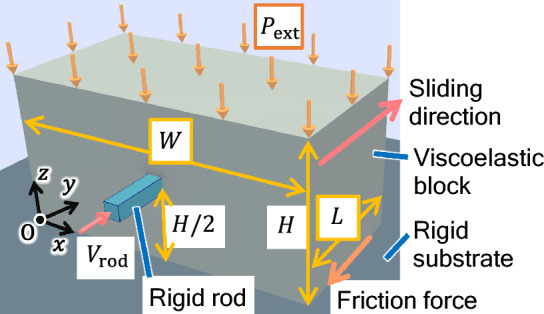
Schematic of a 3D viscoelastic block on a fixed rigid substrate.

We numerically investigate a viscoelastic block on a rigid substrate with width *W*, length *L*, and height *H* along the *x*-, *y*-, and *z*-axes, respectively, as shown in Fig. 1 (see Methods for details). The area of the frictional interface is denoted by $A_0 = L W$. The density, Young’s modulus, and Poisson’s ratio of the block are denoted by $\rho$, *E*, and $\nu$, respectively. The dissipation in the block is characterized by two viscosity coefficients: $\eta _1$ and $\eta _2$. We assume that Amontons’ law holds locally at the interface between the block and the rigid substrate ($z=0$), and the magnitude of the local frictional stress, $\sigma ^{(\text{fric})}(x, y)$ in the interface is locally determined as

1 $$\begin{aligned} \sigma ^{(\text{fric})}(x, y) = \mu (v(x, y)) p(x, y), \end{aligned}$$

where *p*(*x*, *y*) is the bottom pressure, and $\mu (v)$ is the friction coefficient, which depends on the magnitude of the local slip velocity *v*(*x*, *y*) when $v(x,y)\ne 0$ ^29^. Here, $\mu (v)$ is characterized by the characteristic velocity of velocity-weakening friction $v_{\rm c}$ and the local static and kinetic friction coefficients denoted by $\mu _{\rm S}$ and $\mu_{\rm K}$ (see Methods). The rigid rod quasi-statically pushes the center of the side surface along the *y* direction. The macroscopic friction force $F_{\rm T}$ is measured as the force on the rigid rod in the *y* direction. The loading force applied to the top of the block is given by $F_{\rm N} = P_{{\rm ext}}A_0$ with the external pressure to the top surface $P_{{\rm ext}}$.

**Figure 2:**
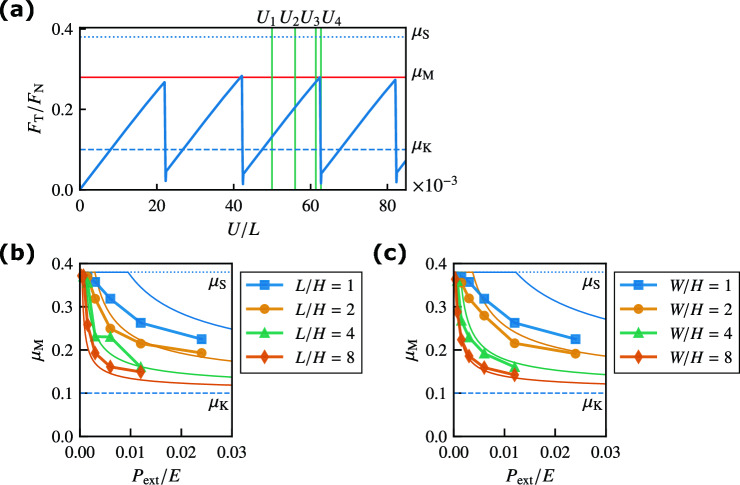
(**a**) Ratio $F_{\rm T}/F_{\rm{N}}$ against the displacement of the rigid rod *U* for $L/H = 1$, $W/H = 2$, and $P_{{\rm ext}}/E = 0.006$. The red horizontal line represents the macroscopic static friction coefficient $\mu _{\rm M}$. (**b**) Macroscopic static friction coefficient $\mu _{\rm M}$ against pressure $P_{{\rm ext}}$ for various *L*/*H* values with $W/H = 1$. The thin solid lines represent the analytical results with $\alpha _{\,\text{A}}= 0.2$ given by Eqs. (4) and (6). (**c**) Macroscopic static friction coefficient $\mu _{\rm M}$ against $P_{{\rm ext}}$ for various *W*/*H* values with $L/H = 1$. The thin solid lines represent the analytical results with $\alpha _{\,\text{B}}= 0.2$ given by Eqs. (6) and (11). The dotted and dashed lines represent $\mu _{\rm S}$ and $\mu _{\rm K}$, respectively.

**Figure 3:**
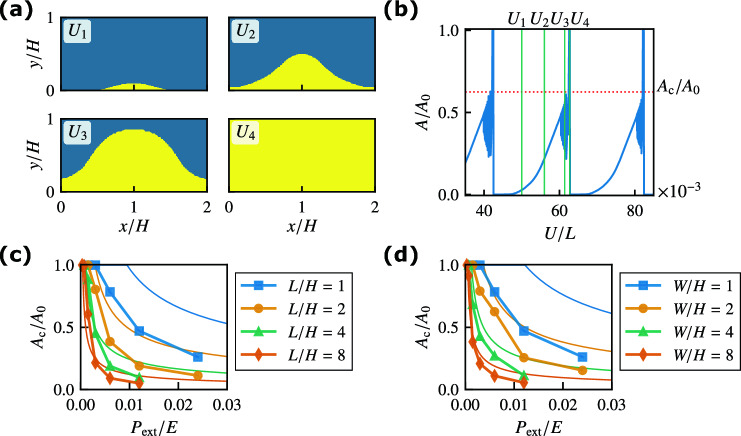
(**a**) Spatial distribution of the slip region in the frictional interface at $U=U_1, U_2, U_3$, and $U_4$ for $L/H = 1$, $W/H = 2$, and $P_{{\rm ext}}/E = 0.006$. The yellow area represents the slip region. The rigid rod is pushing the block at $(x/H,y/H)=(1,0)$. (**b**) Normalized precursor slip area $A/A_0$ against displacement *U*. The dotted line represents the normalized critical area $A_{\rm c}/A_0$. (**c**) Normalized critical area $A_{\rm c}/A_0$ against pressure $P_{{\rm ext}}$ for various *L*/*H* values with $W/H = 1$. The thin solid lines represent the analytical results with $\alpha _{\,\text{A}}= 0.2$ given by Eq. (4). (**d**) Normalized critical area $A_{\rm c}/A_0$ against $P_{{\rm ext}}$ for various *W*/*H* values with $L/H = 1$. The thin solid lines represent the analytical results with $\alpha _{\,\text{B}}= 0.2$ given by Eq. (11).

The ratio $F_{\rm T}/F_{\rm N}$ is plotted against the displacement of the rigid rod *U* for $L/H = 1$, $W/H = 2$, and $P_{{\rm ext}}/E = 0.006$ in Fig. 2a. First, $F_{\rm T}/F_{\rm N}$ increases linearly with *U*. After obtaining a maximum value lower than $\mu _{\rm S}$, $F_{\rm T}/F_{\rm N}$ rapidly decreases to a value close to $\mu _{\rm K}$. This rapid drop is associated with bulk sliding. The significant drop after the linear increase periodically repeats itself. This periodic behavior corresponds to the stick-slip motion of the object. The maximum value of $F_{\rm T}/F_{\rm N}$ represents the macroscopic static friction coefficient, $\mu _{\rm M}$. Figure 2b and c display the macroscopic static friction coefficient $\mu _{\rm M}$ against pressure $P_{{\rm ext}}$ for various *L*/*H* and *W*/*H* values, respectively. The magnitude of $\mu _{\rm M}$ decreases with increasing $P_{{\rm ext}}$, which is qualitatively consistent with the results for a system with a 1D friction interface^10^. The previous study reported the size dependence of $\mu _{\rm M}$ while maintaining the aspect ratio $L/H=2$^10^, whereas Fig. 2b and c demonstrate that the friction coefficient $\mu _{\rm M}$ also decreases with increasing aspect ratios *L*/*H* and *W*/*H*. These results indicate that Amontons’ law breaks down in systems with 2D interfaces.

Figure 3a shows the spatial distribution of the slip region with nonzero slip velocity in the frictional interface at $z=0$ for $U=U_1, U_2, U_3$, and $U_4$ shown in Fig. 2a. Here, we choose $U_1/L=50\times 10^{-3}$, $U_2/L=56\times 10^{-3}$, $U_3/L=61.38\times 10^{-3}$, and $U_4/L=62.71\times 10^{-3}$, which corresponds to the stationary stick-slip region. See Methods for the definition of the slip region. In Fig. 3a, the local precursor slip starts from the region under the rigid rod for $U=U_1$. As *U* increases ($U_2$ and $U_3$), the region expands gradually. After $U=U_3$, the entire area slips with $v>v_{\rm c}$, resulting in bulk sliding. Note that the slip occurs almost along the *y* direction. Figure 3b shows the area of precursor slip *A* normalized by the area of frictional interface $A_0$ against displacement *U*. First, the area of the precursor slip increases gradually with displacement *U*. When the area *A* reaches the critical area $A_{\rm c}$ just before bulk sliding (dotted line), the propagation speed of the area suddenly increases. Owing to rapid propagation, *A* reaches $A_0$ and then returns to 0. We demonstrate the normalized critical area $A_{\rm c}/A_0$ against pressure $P_{{\rm ext}}$ in Fig. 3c and d for various *L*/*H* values with $W/H=1$ and for various *W*/*H* values with $L/H=1$, respectively. The normalized critical area $A_{\rm c}/A_0$ decreases as $P_{{\rm ext}}$, *L*/*H*, or *W*/*H* increases. This decrease is similar to that of $\mu _{\rm M}$ in Fig. 2b and c, respectively.

**Figure 4:**
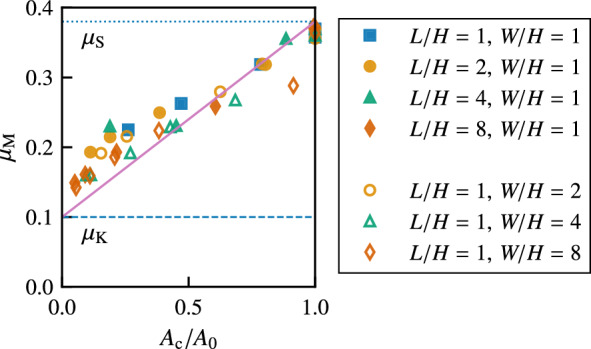
Macroscopic static friction coefficient $\mu _{\rm M}$ against the normalized critical area $A_{\rm c}/A_0$ for various *L*/*H* and *W*/*H* values. The solid line represents the analytical result given by Eq. (6). The dotted and dashed lines represent $\mu _{\rm S}$ and $\mu _{\rm K}$, respectively.

In Fig. 4, we present the macroscopic friction coefficient $\mu _{\rm M}$ against the normalized critical area $A_{\rm c}/A_0$ for various *L*/*H* and *W*/*H* values. The macroscopic friction coefficient $\mu _{\rm M}$ for different *L*/*H* and *W*/*H* values approximately collapses onto a master curve, which indicates a linear increase in $\mu _{\rm M}$ with $A_{\rm c}/A_0$. The minimum value close to $A_{\rm c}/A_0 = 0$ is almost equal to $\mu _{\rm K}$, whereas the maximum value at $A_{\rm c}/A_0=1$ is equal to $\mu _{\rm S}$.

**Figure 5:**
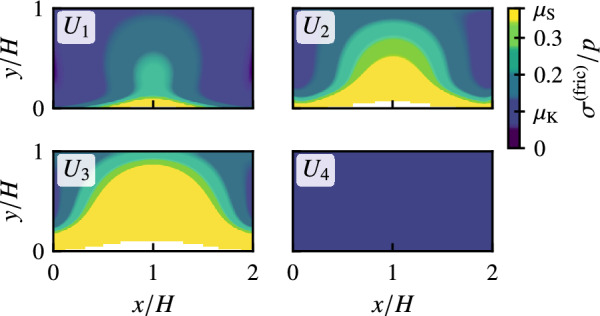
Spatial distribution of the ratio of frictional stress to bottom pressure $\sigma ^{(\text{fric})}/p$ in the frictional interface for $L/H = 1$, $W/H = 2$, and $P_{{\rm ext}}/E = 0.006$ at $U=U_1, U_2, U_3$, and $U_4$. The rigid rod is pushing the block at $(x/H,y/H)=(1,0)$. The white area represents the region with $p=0$ due to the lift of the bottom.

Figure 5 shows the spatial distribution of the ratio $\sigma ^{(\text{fric})}/p$ in the frictional interface for $L/H = 1$, $W/H = 2$, and $P_{{\rm ext}}/E = 0.006$ at $U= U_1, U_2, U_3$, and $U_4$. It should be noted that the direction of the frictional stress is almost opposite to the driving direction, that is, the *y* direction. In the no-slip region, the local static friction can take any value for $0<\sigma ^{(\text{fric})}/p<\mu _{\rm S}$. Before the onset of precursor slip, that is, just after bulk sliding, $\sigma ^{(\text{fric})}/p$ takes a value almost equal to $\mu _{\rm K}$, the local kinetic friction coefficient, in the entire interface, as explained below. At $U=U_1$, $\sigma ^{(\text{fric})}/p$ reaches the local static friction coefficient, $\mu _{\rm S}$, near the rigid rod at $(x/H,y/H)=(1,0)$. As the displacement *U* increases to $U_2$ and $U_3$, the area with $\sigma ^{(\text{fric})}/p \simeq \mu _{\rm S}$ gradually increases. The region of $\sigma ^{(\text{fric})}/p \simeq \mu _{\rm S}$ coincides with the local precursor slip region in Fig. 3a. Except for the slip region, $\sigma ^{(\text{fric})}/p$ remains approximately at $\mu _{\rm K}$. Immediately after $U_3$, bulk sliding with $v>v_{\rm c}$ occurs, and the fast slip leads to $\sigma ^{(\text{fric})}/p = \mu _{\rm K}$ at $U_4$. Bulk sliding rapidly decelerates, and the slip velocity *v* decreases to 0, when $\sigma ^{(\text{fric})}/p$ increases to $\mu _{\rm S}$ in the frictional interface. However, the internal deformation is not able to follow the rapid change, and the ratio of static frictional stress to bottom pressure finally returns to $\sigma ^{(\text{fric})}/p \simeq \mu _{\rm K}$ after bulk sliding. Consequently, $\sigma ^{(\text{fric})}/p$ is almost equal to $\mu _{\rm K}$ after bulk sliding. The macroscopic static friction coefficient $\mu _{\rm M}$ is approximately expressed by the average of $\sigma ^{(\text{fric})}/p$ over the entire frictional interface at $U_3$ immediately before bulk sliding. This result explains the dependence of $\mu _{\rm M}$ on $A_{\rm c}/A_0$ shown in Fig. 4, where $\mu _{\rm M}$ approaches $\mu _{\rm S}$ for $A_{\rm c}/A_0=1$.

### Analysis based on simplified models

To theoretically analyze the numerical results, we employ two simplified models, which explain the dependence of $\mu _{\rm M}$ on *L*/*H* and *W*/*H* (see Supplementary Note online for details).

**Figure 6:**
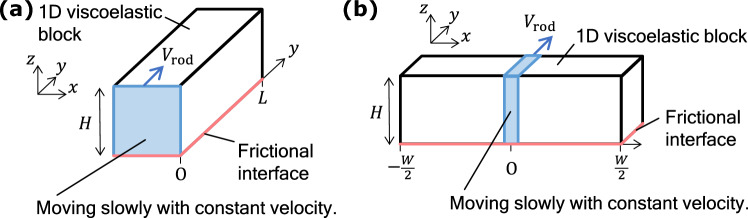
Schematics of simplified models for (**a**) $L/H \gg 1$ and (**b**) $W/H \gg 1$.

#### Model for large *L*/*H*

To discuss the behavior of increasing *L*/*H* while maintaining $W/H=1$, we employ a 1D effective model, as shown in Fig. 6a. The slip region propagates along the *y* direction, as shown in the Supplementary Note and Supplementary Video S1. Therefore, in this model, the degrees of freedom in the *z* and *x* directions are neglected by assuming $W/H\ll L/H$, and the deformation is characterized only by the *y*-dependent displacement in the *y* direction, $u_y(y, t)$, at the interface $z=0$. We also assume a uniform bottom pressure $P_{{\rm ext}}$. The equation of motion is given by

2 $$\begin{aligned} \rho \ddot{u}_y(y,t) = \frac{\partial \sigma _{yy}(y,t)}{\partial y} - \frac{\mu ( {\dot{u}}_y(y,t) ) P_{{\rm ext}}}{\alpha _{\,\text{A}}H}, \end{aligned}$$

where $\ddot{u}_y$ and ${\dot{u}}_y$ are the second- and first-order time derivatives of the displacement, respectively. Here, $\alpha _{\,\text{A}}$ represents the effect of the block thickness and is treated as a fitting parameter. The normal stress $\sigma _{yy}$ is given by

3 $$\begin{aligned} \sigma _{yy}(y,t) = E_1 \frac{\partial u_y}{\partial y} + \eta _{\,{\rm T}}\frac{\partial {\dot{u}}_y}{\partial y} \end{aligned}$$

with the elastic constant $E_1 = E/\left\{ (1+\nu )(1-\nu ) \right\}$ and viscous constant $\eta _{\,{\rm T}}= \eta _1 (\eta _1 + 2 \eta _2)/(\eta _1 + \eta _2)$ in the plane stress state by considering the block as a thin plate (see Methods).

The quasi-static solution $u^{\mathrm {(a)}}_{y}(y)$ of Eq﻿s. (2) and (3) with $\ddot{u}_y={\dot{u}}_y=0$ is obtained analytically, where the precursor slip area *A* increases with *U* (see Supplementary Note). A linear stability analysis reveals that the quasi-static solution becomes unstable, and bulk sliding occurs when *A* reaches the critical area $A_{\rm c}$ owing to the competition between velocity-weakening friction and viscosity. The critical area $A_{\rm c}$ satisfies

4 $$\begin{aligned} \pi ^2 \eta _{\,{\rm T}}\left( \frac{A_{\rm c}}{A_0} \right) ^{-2} + 2 \pi L \sqrt{ \rho E_1 } \left( \frac{A_{\rm c}}{A_0} \right) ^{-1} = \frac{\left( \mu _{\rm S}- \mu _{\rm K}\right) P_{{\rm ext}}L^2}{v_{\rm c}\alpha _{\,\text{A}}H} \end{aligned}$$

(see Supplementary Note). For $A_{\rm c}/A_0 \ll 1$, Eq. (4) yields

5 $$\begin{aligned} \frac{A_{\rm c}}{A_0} \simeq \pi \left( \frac{\mu _{\rm S}- \mu _{\rm K}}{\alpha _{\,\text{A}}} \right) ^{-\frac{1}{2}} \left( \frac{P_{{\rm ext}}H}{\eta _{\,{\rm T}}v_{\rm c}} \right) ^{-\frac{1}{2}} \left( \frac{L}{H} \right) ^{-1}. \end{aligned}$$

This equation indicates that the normalized critical area $A_{\rm c}/A_0$ decreases as *L*/*H* or $P_{{\rm ext}}$ increases, which is consistent with the FEM results shown in Fig. 3c. We plot $A_{\rm c}/A_0$ obtained from Eq. (4) as thin solid lines in Fig. 3c by choosing the fitting parameter $\alpha _{\,\text{A}}= 0.2$ to match the results of the FEM simulations. The analytical results semi-quantitatively reproduce the numerical results except for $L/H=1$.

The quasi-static solution $u^{\mathrm {(a)}}_{y}(y)$ yields

6 $$\begin{aligned} \mu _{\rm M}= \mu _{\rm K}+ (\mu _{\rm S}- \mu _{\rm K}) \frac{A_{\rm c}}{A_0}. \end{aligned}$$

This is consistent with the FEM simulations, as shown by the solid line in Fig. 4. For $A_{\rm c}/A_0 \ll 1$, substituting Eq. (5) into this equation, we obtain

7 $$\begin{aligned} \mu _{\rm M}- \mu _{\rm K}\simeq \pi \left( \mu _{\rm S}- \mu _{\rm K}\right) ^{\frac{1}{2}} \alpha _{\,\text{A}}^{\frac{1}{2}} \left( \frac{P_{{\rm ext}}H}{\eta _{\,{\rm T}}v_{\rm c}} \right) ^{-\frac{1}{2}} \left( \frac{L}{H} \right) ^{-1}. \end{aligned}$$

This equation indicates that the macroscopic static friction coefficient $\mu _{\rm M}$ decreases as $P_{{\rm ext}}$ or *L*/*H* increases. We plot $\mu _{\rm M}$ given by Eqs. (4) and (6) as thin solid lines in Fig. 2b, which semi-quantitatively reproduces the results of the FEM simulations except for $L/H=1$.

In a previous study^10^, $\mu _{\rm M}$ is obtained analytically as

8 $$\begin{aligned} \mu _{\rm M}- \mu _{\rm K}\simeq \frac{\pi ^2}{\pi ^2 - 4} \left( \mu _{\rm S}- \mu _{\rm K}\right) ^{\frac{2}{3}} \alpha ^{\frac{1}{3}} \left( \frac{P_{{\rm ext}}H}{\eta _{\,{\rm T}}v_{\rm c}} \right) ^{-\frac{1}{3}} \left( \frac{L}{H} \right) ^{-\frac{2}{3}} \end{aligned}$$

for $A_{\rm c}/A_0 \ll 1$ in a system with small *L*/*H*. Here, $\alpha$ is the fitting parameter. The power-law exponents in Eq. (8) for the dependence on $P_{{\rm ext}}$ and *L*/*H* differ from those in Eq. (7). The present model assumes $L/H\gg 1$, which results in a uniform bottom pressure, as shown in the Supplementary Note. For a small *L*/*H*, the bottom pressure increases along the driving direction owing to the torque effect^10,21^, and the analytical results deviate from those of FEM due to the non-uniform pressure as shown in Figs. 2b and 3c, which leads to different exponents from those in the present study.

#### Model for large *W*/*H*

To discuss the behavior of increasing *W*/*H* while maintaining $L/H=1$, we employ a 1D effective model, as shown in Fig. 6b. For $W/H \gg 1$, the slip region propagates along the *x* direction, as shown in Supplementary Note and Supplementary Video S2. Hence, in this model, we characterize the deformation only by the *x*-dependent displacement in the *y* direction, $u_y(x, t)$, at the interface $z=0$ by assuming $L/H\ll W/H$. We also assume the rod size is sufficiently small and negligible. The equation of motion is given by

9 $$\begin{aligned} \rho \ddot{u}_y(x,t) = \frac{\partial \sigma _{xy}(x,t)}{\partial x} - \frac{ \mu ({\dot{u}}_y(x, t)) P_{{\rm ext}}}{ \alpha _{\,\text{B}}H }. \end{aligned}$$

Here, $\alpha _{\,\text{B}}$ represents the effect of the block thickness and is treated as a fitting parameter. The shear stress $\sigma _{xy}$ is given by

10 $$\begin{aligned} \sigma _{xy} = E_2 \frac{\partial u_y}{\partial x} + \frac{\eta _1}{2} \frac{\partial {\dot{u}}_y}{\partial x} \end{aligned}$$

with the elastic constant $E_2 = E/\left\{ 2(1+\nu )\right\}$ and the viscous constant $\eta _1/2$ (see Methods).

The quasi-static solution $u^{\mathrm {(a)}}_{y}(x)$ is also obtained analytically, where the precursor slip area *A* increases with the value of *U* (see Supplementary Note). The linear stability analysis reveals that the precursor slip becomes unstable, and bulk sliding occurs when *A* reaches the critical area $A_{\rm c}$ satisfying

11 $$\begin{aligned} 2 \pi ^2 \eta _1 \left( \frac{A_{\rm c}}{A_0} \right) ^{-2} + 4 \pi W \sqrt{ \rho E_2 } \left( \frac{A_{\rm c}}{A_0} \right) ^{-1} = \frac{\left( \mu _{\rm S}- \mu _{\rm K}\right) P_{{\rm ext}}W^2 }{ v_{\rm c}\alpha _{\,\text{B}}H }. \end{aligned}$$

For $A_{\rm c}/A_0 \ll 1$, this equation yields

12 $$\begin{aligned} \frac{A_{\rm c}}{A_0} \simeq \pi \left( \frac{\mu _{\rm S}- \mu _{\rm K}}{\alpha _{\,\text{B}}} \right) ^{-\frac{1}{2}} \left( \frac{P_{{\rm ext}}H}{2 \eta _1 v_{\rm c}} \right) ^{-\frac{1}{2}} \left( \frac{W}{H} \right) ^{-1}. \end{aligned}$$

The power-law exponents for the pressure and aspect ratio are the same as those in Eq. (5). This equation indicates that $A_{\rm c}/A_0$ decreases as $P_{{\rm ext}}$ or *W*/*H* increases. We plot $A_{\rm c}/A_0$ given by Eq. (11) as thin solid lines in Fig. 3d, which semi-quantitatively reproduces the results of the FEM analysis by choosing $\alpha _{\,\text{B}}=0.2$ except for $W/H=1$. For small *W*/*H*, the size of the rod and the *y*-dependence of the displacement become relevant, which leads to the deviation between the numerical and theoretical results.

The macroscopic static friction coefficient $\mu _{\rm M}$ is given by Eq. (6). For $A_{\rm c}/A_0 \ll 1$, substituting Eq. (12) into Eq. (6), we obtain

13 $$\begin{aligned} \mu _{\rm M}- \mu _{\rm K}\simeq \pi \left( \mu _{\rm S}- \mu _{\rm K}\right) ^{\frac{1}{2}} \alpha _{\,\text{B}}^{\frac{1}{2}} \left( \frac{P_{{\rm ext}}H}{2 \eta _1 v_{\rm c}} \right) ^{-\frac{1}{2}} \left( \frac{W}{H} \right) ^{-1}. \end{aligned}$$

The macroscopic static friction coefficient $\mu _{\rm M}$ decreases as $P_{{\rm ext}}$ or *W*/*H* increases. The thin solid lines shown in Fig. 2c are given by Eqs. (6) and (11), and they semi-quantitatively reproduce the results of the FEM simulations except for $W/H=1$.

## Discussion

In this study, we numerically investigate the sliding motion of a 3D viscoelastic object using the FEM. The critical area of the precursor slip and macroscopic static friction coefficient decrease with an increase in the external pressure, length, or width of the object. The analysis based on the simplified models reveals that the stability condition determines the critical area of the precursor slip owing to the competition between the velocity-weakening friction and viscosity. The analysis explains the dependence of macroscopic static friction in the FEM simulations.

In a previous study^10^, the aspect ratio of the system is fixed at $L/H=2$ to investigate the size and load dependences of the precursor slip and the breakdown of Amontons’ law. For $L/H=2$, the nonuniformity of the bottom pressure is remarkable, which is considered to be the origin of the precursor slip and the breakdown of Amontons’ law. However, the present results with various aspect ratios show that the nonuniformity of shear stress also causes these behaviors without non-uniform pressure. Although the model considered in the previous study reproduces the results of systems with a smaller *L*/*H* better, the simplified model in this study is more appropriate for systems with a large *L*/*H* (see Supplementary Note).

The parameters for the FEM simulations employed here are those of a virtual material, and different from those of poly methyl methacrylate (PMMA) employed in experiments^12,15^. We choose them to compare our results with the 2D simulations of previous studies^10^ and to reduce the computational load (see Methods). It also should be noted that the driving rod employed in experiments is hard but has finite stiffness, which is different from the rigid rod used in this study. The effect of the finite stiffness of the driving rod is considered to be small because it is taken into account as a deformation of the viscoelastic block around the driving point. In addition, we have ignored the aging effect^7^ in the local friction model because a previous experiment using PMMA^15^ indicates that the time scale of the aging is larger than that of the stick of the macroscopic stick-slip motion. The difference in the parameters, the driving method, and the local friction model may affect our results. However, FEM simulations employing similar parameters semi-quantitatively reproduce the external pressure dependence of the macroscopic static friction coefficient obtained in the experiment using PMMA^12^. The dependence of the macroscopic static friction coefficient on the aspect ratio for PMMA is also considered to be consistent with our present results. The dependence on material parameters, the driving methods, and the local friction model will be investigated in future work.

The dependence of the static and kinetic friction coefficients on the pressure or block shape has been studied in experiments using rubber blocks^30–32^. The results of these experiments are partially consistent with ours, but there is a difference in the dependence on the aspect ratio. In these experiments, the methods to change the aspect ratio and drive the block differ from those used in this study. For the rubber block, the local Amontons’ law used in this study may not be applicable because the real contact area can become comparable to the apparent contact area, which contradicts the assumption of the Amontons’ law. We need further investigations to determine the origin of the difference.

Recent numerical simulations of spring-block models have shown that the friction coefficient changes with the geometric pattern of the frictional interface^33–36^. However, our results indicate that an object shape can also control the macroscopic static friction coefficient. This might lead to new insights into methods for controlling friction in various objects, including shoe soles and tires.

Precursor slip has been investigated experimentally for the sliding motion of PMMA blocks based on fracture mechanics^26,37–41^. Such a precursor slip is related to pre-earthquakes that occur a few days or months before a major earthquake^42–44^, which are studied using frictional spring-block models^45^. However, these studies have focused on 1D frictional interfaces or discrete models, which differ from 2D friction interfaces in more realistic systems. Our results for a 3D system with a 2D interface will provide new insights into the precursor slip observed in realistic situations.

## Methods

### Setting of system

The equation of motion for a viscoelastic body is given by

14 $$\begin{aligned} \rho \ddot{{\varvec{u}}} = {\varvec{\nabla }} \cdot {\varvec{\sigma }} \end{aligned}$$

with displacement ${\varvec{u}}$, stress ${\varvec{\sigma }}$, and second-order time derivative $\ddot{{\varvec{u}}}$ of displacement. The stress ${\varvec{\sigma }}$ is given by the sum of the elastic stress ${\varvec{\sigma }}^\mathrm {(E)}$ obeying Hooke’s law and the viscous stress ${\varvec{\sigma }}^\mathrm {(V)}$, which is proportional to the strain rate. We assume that the viscoelastic body is isotropic. The elastic stress tensor $\sigma ^\mathrm {(E)}_{ij}$ is given by

15 $$\begin{aligned} \sigma ^\mathrm {(E)}_{ij} = \frac{E}{1+\nu } \,\varepsilon _{ij} + \frac{\nu E}{(1+\nu )(1-2\nu )} \,\varepsilon _{kk} \delta _{ij} \end{aligned}$$

with the Kronecker delta $\delta _{ij}$ and the strain tensor $\varepsilon _{ij}$. The viscous stress tensor $\sigma ^{ ( \text{V} ) }_{ij}$ is given by

16 $$\begin{aligned} \sigma ^\mathrm {(V)}_{ij} = \eta _1\,\dot{\varepsilon }_{ij} + \eta _2\,\dot{\varepsilon }_{kk} \delta _{ij} \end{aligned}$$

with the strain rate tensor $\dot{\varepsilon }_{ij}$^46^. The boundary conditions for the top surface at $z=H$ are $\sigma _{zz} = -P_{{\rm ext}}$ and $\sigma _{zx} = \sigma _{zy} = 0$. At the free surface for $x=0,W$ or $y=0,L$, we assume ${\varvec{\sigma }} \cdot {\varvec{n}} = {\varvec{0}}$ with the normal vector ${\varvec{n}}$ of the surface. The boundary conditions at the contact surface with a rigid rod ($y=0$) are given by $\sigma _{yx} = \sigma _{yz} = 0$ and ${\dot{u}}_y = V_{\rm{rod}}$, where ${\dot{u}}_y$ is the velocity in the *y* direction and $V_{\rm{rod}}$ is the velocity of rigid rod. At the bottom of the block ($z=0$) in contact with a rigid substrate, the bottom pressure $p = -\sigma _{zz}$ is determined such that the displacement $u_z$ in the *z* direction is 0. However, the bottom pressure is limited to $p \ge 0$. The region of the bottom surface with $u_z > 0$ and $p=0$ becomes a free surface with ${\varvec{\sigma }} \cdot {\varvec{n}} = {\varvec{0}}$. The boundary condition in the tangential direction at the bottom with $p>0$ is given by

17 $$\begin{aligned} {\varvec{t}} = -{\varvec{v}}/v ~ \mu (v)p \end{aligned}$$

with the tangential stress vector ${\varvec{t}}(x,y) = (\sigma _{zx},\sigma _{zy})$, local slip velocity vector ${\varvec{v}}(x,y) = ({\dot{u}}_x, {\dot{u}}_y)$, velocity ${\dot{u}}_x$ in the *x* direction, and velocity ${\dot{u}}_y$ in the *y* direction. The direction of the frictional stress is opposite to that of the local slip velocity. Frictional stress is defined as $\sigma ^{(\text{fric})}(x,y) = |{\varvec{t}}|$. The slip velocity is defined as $v(x,y) = |{\varvec{v}}(x,y) |$.

The frictional stress $\sigma ^{(\text{fric})}$ is given by Eq. (1). In the case $v(x,y) = 0$, the frictional stress is balanced with the local shear stress, where the maximum magnitude of the former is given by $\mu _{\rm S}p(x, y)$. The local friction coefficient $\mu (v)$ linearly decreases from $\mu _{\rm S}$ to $\mu _{\rm K}$ for $0 < v \le v_{\rm c}$ and $\mu _{\rm K}$ for $v > v_{\rm c}$. Amontons’ law is expected to hold locally if the local region considered in the frictional interface contains a sufficiently large number of real contact points and has negligibly small spatial variations in internal stress^8,9,47^.

To treat static friction in the numerical simulation, we introduce a small velocity scale $v_\text{e}$. The local friction coefficient $\mu (v)$ is given by

18 $$\begin{aligned} \mu (v) = \left\{ \begin{array}{lll} \mu _{\rm S}\,v/v_\text{e}, ~&{} 0 \le v \le v_\text{e} \\ \mu _{\rm S}- \left( \mu _{\rm S}-\mu _{\rm K}\right) v/v_{\rm c}, ~&{} v_\text{e}< v < v_{\rm c}\\ \mu _{\rm K}, ~&{} v \ge v_{\rm c}\end{array} \right. ~. \end{aligned}$$

We consider the limit $v_\text{e} \rightarrow +0$. The region with $0 \le v \le v_\text{e}$ corresponds to static friction. The slip area *A* is defined as the region with $v > v_\text{e}$.

### Details of 3D FEM simulation

The viscoelastic block is divided into cubes with length $\Delta x$ consisting of six tetrahedra. The displacements and velocities within each element are approximated using a linear interpolation. We choose the characteristic velocity $v_\text{e}/V_{\rm{rod}}= 2.5\times 10 ^{-2}$ such that $v_\text{e}/V_{\rm{rod}}\ll 1$ is satisfied. In the FEM simulations, we select $\Delta x/H=1/40$, $\Delta t/( H \sqrt{\rho/E} )\thickapprox 10^{-6}$, where $\Delta t$ is a time step, and $V_{\rm{rod}} \sqrt{\rho/E}=2.83\times 10^{-5}$. We have confirmed that the numerical results do not change, even if we use smaller values.

First, we apply an external uniform pressure $P_{{\rm ext}}$ to the top surface and relax the system to an equilibrium state. After relaxation, the center of the side surface $(x,y,z)=(W/2, 0, H/2)$ is pushed along the *y* direction by a rigid rod from time $t=0$ with a sufficiently slow speed $V_{\rm{rod}}$. The displacement of the rigid rod is denoted by $U(t)= V_{\rm{rod}}t$. The length of one side of a rigid square rod is 0.1*H*, and the height of its center from the bottom is 0.5*H*.

### Details of analysis based on simplified models

**Model for large** *L*/*H*: The second term on the right-hand side of Eq. (2) represents local friction. Here, we assume a constant bottom pressure given by $P_{{\rm ext}}$, which is verified in the FEM simulations for $L/H \gg 1$ as shown in the Supplementary Note and Supplementary Video S1. The local friction coefficient $\mu$ is expressed as a function of $v=|{\dot{u}}_y|$. Note that $0 \le \mu \le \mu _{\rm S}$ when $v=0$. The boundary conditions are $\partial u_y(y=L, t)/\partial y = 0$ and $u_y(y=0, t) = U(t)$. In our analysis, we set the origin of *U* immediately after the bulk sliding and assume that the ratio of the frictional stress to $P_{{\rm ext}}$ is equal to $\mu _{\rm K}$ at $U=0$.

**Model for large** *W*/*H*: The second term on the right-hand side of Eq. (9) represents the friction. The bottom pressure is almost independent of *x* in the FEM simulations, as shown in the Supplementary Note and Supplementary Video S2. Therefore, we assume a constant bottom pressure given by $P_{{\rm ext}}$. The boundary conditions are $\partial u_y (|x|=W/2, t)/\partial x = 0$ and $u_y(x=0, t) = U(t)$.

### Parameters

The parameters for the viscoelastic object are chosen as $\nu=0.34$, $\eta_1/( H \sqrt{\rho E} ) = 1.41$, and $\eta_2/\eta_1=1$, whereas we set the parameters for the friction as $\mu_\text S=0.38$, $\mu_\text K=0.1$, and $v_\text c \sqrt{\rho / E} = 4.81\times10^{-4}$, following previous FEM simulations^10^. These values are different from those adopted for the experiment using PMMA^10,12^. The parameters for the PMMA blocks^12^ are estimated as $L/H=5$, $W/H = 0.25$, $P_{{\rm ext}}/E \thickapprox 3 \times 10^{-4}$, $\nu = 0.4$, $\mu _{\rm S}= 1.2$, and $\mu _{\rm K}= 0.2$, and much smaller $v_{\rm c}\sqrt{\rho /E }$ and $\eta _1 / (H \sqrt{\rho E})$ are used in the previous study^10^.

### Supplementary Information

Supplementary Information 1.

Supplementary Information 2.

Supplementary Information 3.

## Data Availability

The datasets used and/or analyzed during the current study available from the corresponding author on reasonable request.
