# Author Correction: Static friction coefficient depends on the external pressure and block shape due to precursor slip

**DOI:** 10.1038/s41598-024-55381-2

**Published:** 2024-03-05

**Authors:** Wataru Iwashita, Hiroshi Matsukawa, Michio Otsuki

**Affiliations:** 1 Department of Mechanical Science and Bioengineering, Osaka University, Toyonaka, 560-8531 Japan; 2 Department of Physical Sciences, Aoyama Gakuin University, Sagamihara, 252-5258 Japan

Correction to: *Scientific Reports* 10.1038/s41598-023-29764-w, published online 13 February 2023

The original version of this Article contained errors in the Methods section, Figures 2 and 3, and Supplementary Figure 3.

In the Methods section, the values of ${V_{{\text{rod}}}}$, ${\eta_1}$, and ${v_\text c}$ were incorrect.

Under the subheading ‘Details of 3D FEM simulation’,

“In the FEM simulations, we select $\Delta x/H = 1/40$, $\Delta t/(H\sqrt {\rho /E} ) \approx {10^{ - 6}}$, and ${{{V}}_{{\text{rod}}}}\sqrt {\rho /E} = 2 \times {10^{ - 5}}.$”

now reads:

“In the FEM simulations, we select $\Delta x/H = 1/40$, $\Delta t/(H\sqrt {\rho /E} ) \approx {10^{ - 6}}$, where $\Delta t$ is a time step, and ${{{V}}_{{\text{rod}}}}\sqrt {\rho /E} = 2.83 \times {10^{ - 5}}.$”

Under the subheading ‘Parameters’,

“The parameters for the viscoelastic object are chosen as $\nu = 0.34, \, {\eta_1}/(H\sqrt {\rho E} ) = 2,{\text{ and }}{\eta_2}/{\eta_1} = 1$, whereas we set the parameters for the friction as ${\mu_\text S} = 0.38,  \mu_{\text{K}} = 0.1,{\text{ and }}{{{v}}_{\text{c}}} \, \sqrt {\rho /E} = 3.4 \times {10^{ - 4}},$ following previous FEM simulations.”

now reads:

“The parameters for the viscoelastic object are chosen as $\nu = 0.34, \, {\eta_1}/(H\sqrt {\rho E} ) = 1.41,{\text{ and }}{\eta_2}/{\eta_1} = 1$, whereas we set the parameters for the friction as ${\mu _\text S} = 0.38, \mu_{\text{K}} = 0.1,{\text{ and }}{{{v}}_{\text{c}}} \, \sqrt {\rho /E} = 4.81 \times {10^{ - 4}},$ following previous FEM simulations.”

The values of ${v_\text{c}}$ and ${\eta_1},$ the thin solid lines in Figure 2 (b), (c) and Figure 3 (c), (d), representing the analytical results, were incorrect.

The original Figures 2 and 3 and accompanying legends appear below.

**Figure 2:**
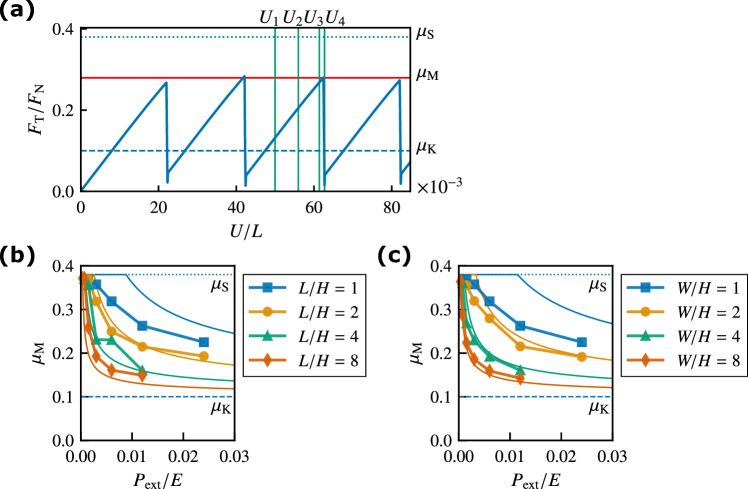
(**a**) Ratio $F_{\rm T}/F_{\rm{N}}$ against the displacement of the rigid rod *U* for $L/H = 1$, $W/H = 2$, and $P_{{\rm ext}}/E = 0.006$. The red horizontal line represents the macroscopic static friction coefficient $\mu _{\rm M}$. (**b**) Macroscopic static friction coefficient $\mu _{\rm M}$ against pressure $P_{{\rm ext}}$ for various *L*/*H* values with $W/H = 1$. The thin solid lines represent the analytical results with $\alpha _{\,\text{A}}= 0.2$ given by Eqs. (4) and (6). (**c**) Macroscopic static friction coefficient $\mu _{\rm M}$ against $P_{{\rm ext}}$ for various *W*/*H* values with $L/H = 1$. The thin solid lines represent the analytical results with $\alpha _{\,\text{B}}= 0.2$ given by Eqs. (6) and (11). The dotted and dashed lines represent $\mu _{\rm S}$ and $\mu _{\rm K}$, respectively.

**Figure 3:**
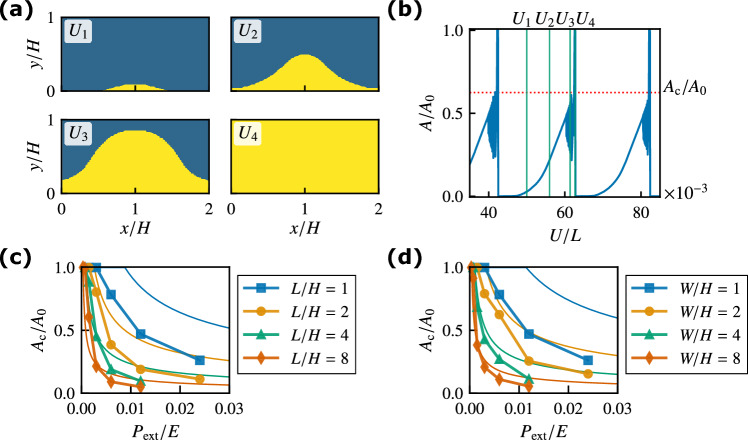
(**a**) Spatial distribution of the slip region in the frictional interface at $U=U_1, U_2, U_3$, and $U_4$ for $L/H = 1$, $W/H = 2$, and $P_{{\rm ext}}/E = 0.006$. The yellow area represents the slip region. The rigid rod is pushing the block at $(x/H,y/H)=(1,0)$. (**b**) Normalized precursor slip area $A/A_0$ against displacement *U*. The dotted line represents the normalized critical area $A_{\rm c}/A_0$. (**c**) Normalized critical area $A_{\rm c}/A_0$ against pressure $P_{{\rm ext}}$ for various *L*/*H* values with $W/H = 1$. The thin solid lines represent the analytical results with $\alpha _{\,\text{A}}= 0.2$ given by Eq. (4). (**d**) Normalized critical area $A_{\rm c}/A_0$ against $P_{{\rm ext}}$ for various *W*/*H* values with $L/H = 1$. The thin solid lines represent the analytical results with $\alpha _{\,\text{B}}= 0.2$ given by Eq. (11).

In addition, the thin lines in the Supplementary Figure 3, representing the analytical results, were incorrect.

The original Article and accompanying Supplementary Information file have been corrected.

### Supplementary Information

Supplementary Information.

