# Supplementary material for: Author Correction: Static friction coefficient depends on the external pressure and block shape due to precursor slip
Source: Sci Rep. 2024 Mar 5;14:5461. doi: 10.1038/s41598-024-55381-2 (PMC10915153; doi:10.1038/s41598-024-55381-2)
Supplement: Supplementary file 1 — Supplementary Information. [file 41598_2024_55381_MOESM1_ESM.pdf]

Static Friction Coefficient Depends on the External Pressure and Block Shape due to Precursor Slip

Wataru Iwashita[1], **Hiroshi Matsukawa**[2], **and Michio Otsuki**[1]

[1] Osaka University, Department of Mechanical Science and Bioengineering, Toyonaka, 560-8531, Japan
[2] Aoyama Gakuin University, Department of Physical Sciences, Sagamihara, 252-5258, Japan

Supplementary Information

Supplementary Video (Titles and legends)

Supplementary Video S1. Spatial distributions of different quantities in the frictional interface for $P_{\mathrm{ext}}/E = 0.012$, $L/H = 4$, and $W/H = 1$.

Left panel shows the ratio $F_{\mathrm{T}}/F_{\mathrm{N}}$ against the displacement of the rigid rod U. (a) Spatial distribution of the slip region, which is represented by the yellow area. (b) Spatial distribution of the ratio $\sigma^{(\mathrm{fric})}/p$. The white area represents the region with $p = 0$ due to the lift at the bottom. (c) Spatial distribution of p.

Supplementary Video S2. Spatial distributions of different quantities in the frictional interface for $P_{\mathrm{ext}}/E = 0.012$, $L/H = 1$, and $W/H = 4$.

Left panel shows the ratio $F_{\mathrm{T}}/F_{\mathrm{N}}$ against U. (a) Spatial distribution of the slip region, which is represented by the yellow area. (b) Spatial distribution of the ratio $\sigma^{(\mathrm{fric})}/p$. The white area represents the region with $p = 0$ due to the lift at the bottom. (c) Spatial distribution of p.

Supplementary Note

In this supplementary note, we provide a detailed description of the analysis presented in the main text. Section 1 describes the FEM results for the spatial distribution of quantities at the frictional interface. Details of the analytical calculation based on the simplified models are presented in Sec. 2. In Sec. 3, we compare our results with those of a previous study[1].

1. Spatial distributions of quantities in the frictional interface

This section presents the spatial distributions of the quantities in the frictional interface for the 3D FEM simulations, which supports the assumption used to derive the simplified models in the main text. The spatial distributions for $L/H = 4$ and $W/H = 1$ are shown in Fig. S1, corresponding to Supplementary Video S1. Figures S1 (a) and (b) demonstrate the spatial distributions of the slip region and the ratio $\sigma^{(\mathrm{fric})}/p$, respectively. Both distributions exhibit a slight dependency on x. Therefore, in the simplified model with $L/H \gg 1$, we neglect the x dependence. The spatial distribution of pressure p at the bottom is shown in Fig. S1 (c). The pressure p is approximately equal to the pressure P_{ext} at the top surface of the entire bottom plane. Based on this numerical result, we assume that the bottom pressure is uniform in the simplified model for $L/H \gg 1$.

The spatial distributions in the frictional interface for $W/H = 4$ with $L/H = 1$ are shown in Fig. S2, corresponding to Supplementary Video S2. Figures S2 (a) and (b) demonstrate the spatial distributions of the slip region and the ratio $\sigma^{(\mathrm{fric})}/p$, respectively. The regions for precursor slip and $\sigma^{(\mathrm{fric})}/p \approx \mu_{\mathrm{S}}$ propagate from the center at $x/H = 2$, which is different from those for $L/H \gg 1$ shown in Fig. S1. The characteristic behavior is the extension of the slip region along the x direction, as shown in Supplementary Video S2. Therefore, in the model for

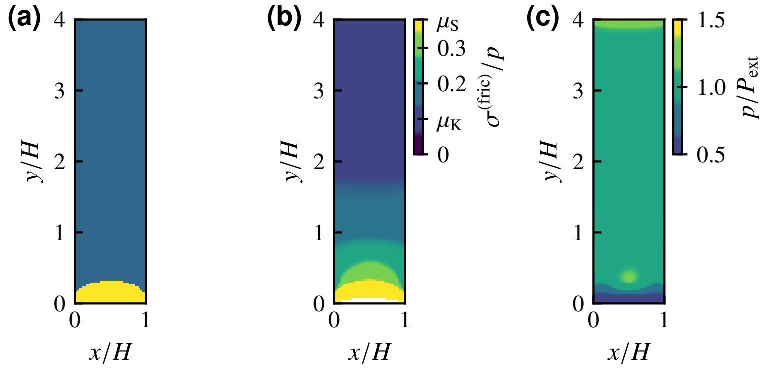

Figure S1. Spatial distributions of different quantities in the frictional interface for $P_{\mathrm{ext}}/E = 0.012$, $L/H = 4$, $W/H = 1$, and $U/L = 2.6 \times 10^{-2}$. The rigid rod pushes the block at $(x/H, y/H) = (0.5, 0)$. (a) Spatial distribution of the slip region, which is represented by the yellow area. (b) Spatial distribution of the ratio $\sigma^{(\mathrm{fric})}/p$. (c) Spatial distribution of p.

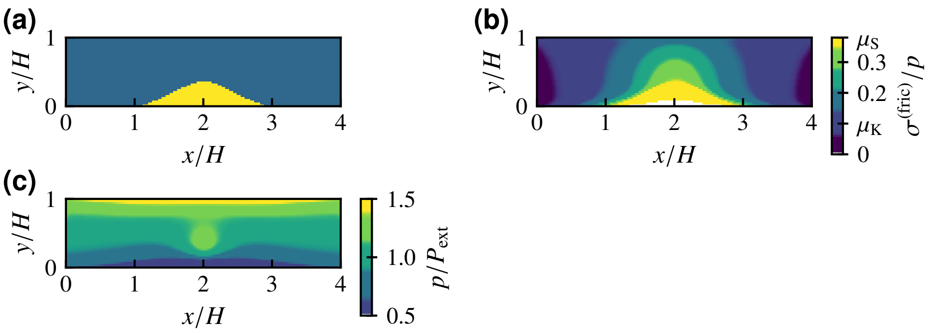

Figure S2. Spatial distributions of different quantities in the frictional interface for $P_{\mathrm{ext}}/E = 0.012$, $L/H = 1$, $W/H = 4$, and $U/L = 1.08 \times 10^{-1}$. The rigid rod pushes the block at $(x/H, y/H) = (2, 0)$. (a) Spatial distribution of the slip region, which is represented by the yellow area. (b) Spatial distribution of the ratio $\sigma^{(\mathrm{fric})}/p$. (c) Spatial distribution of p.

$W/H \gg 1$, we neglect the dependence on y. The spatial distribution of pressure p at the bottom is shown in Fig. S2 (c). The variation in p is not strong. Therefore, we assume a constant pressure in the simplified model for $W/H \gg 1$.

2. Analysis based on the simplified models

This section presents details of the analytical calculations based on the simplified models. We derive the results for $L/H \gg 1$ and $W/H \gg 1$ shown in Secs. 2.1 and 2.2.

2.1. Model for large L/H

Quasi-static solution

The quasi-static solution $u_{y0}(y)$ of equations (2) and (3) in the main text with the local friction coefficient $\mu = \mu_{\mathrm{K}}$ and acceleration $\ddot{u}_y(y,t) = 0$ at $U = 0$ is given by

$$u_{y0}(y) = \frac{\mu_{\mathrm{K}} P_{\mathrm{ext}}}{E_1 \alpha_{\mathrm{A}} H} \left(\frac{y^2}{2} - Ly \right). \tag{S1}$$

The quasi-static solution $u_y^{(\mathrm{a})}(y)$ for $U > 0$ is given by

$$u_y^{(\mathrm{a})}(y) = \begin{cases} u_{y1}(y), & 0 \le y \le l \\ u_{y0}(y), & l < y \le L \end{cases}, \tag{S2}$$

where l is the length of the precursor slip. The slip area A is given by

$$A = lW. \tag{S3}$$

Here, we set the local friction coefficient μ to be equal to μ_S for $0 \le y \le l$, and the displacement and stress are continuous at $y = l$:

$$u_{y1}(y=l) = u_{y0}(y=l) \tag{S4}$$
$$\left.\frac{du_{y1}}{dy}\right|_{y=l} = \left.\frac{du_{y0}}{dy}\right|_{y=l}, \tag{S5}$$

which gives

$$u_{y1}(y) = u_{y0}(y) + \frac{(\mu_S - \mu_K) P_{ext}}{2E_1 \alpha_A H}\left(y^2 - 2ly + l^2\right) \tag{S6}$$

and

$$l = \left\{\frac{2E_1 \alpha_A H\, U}{(\mu_S - \mu_K) P_{ext}}\right\}^{\frac{1}{2}}. \tag{S7}$$

From equations (S3) and (S7), A is obtained as

$$\frac{A}{W} = \left\{\frac{2E_1 \alpha_A H\, U}{(\mu_S - \mu_K) P_{ext}}\right\}^{\frac{1}{2}}. \tag{S8}$$

Stability analysis

We introduce the fluctuation $\delta u_y(y,t)$ as

$$\delta u_y(y,t) = u_y(y,t) - u_y^{(a)}(y). \tag{S9}$$

For $y > l$, we assume $\delta u_y(y,t) = 0$. Substituting equation (S9) into the equation of motion (2), we obtain

$$\rho \delta \ddot{u}_y = E_1 \frac{\partial^2 \delta u_y}{\partial y^2} + \eta_t \frac{\partial^2 \delta \dot{u}_y}{\partial y^2} + \frac{(\mu_S - \mu_K)\, \delta \dot{u}_y\, P_{ext}}{v_c \alpha_A H}. \tag{S10}$$

Because $\delta u_y = 0$ at $y = 0$ and l, $\delta u_y(y,t)$ can be expressed as

$$\delta u_y(y,t) = \sum_{m=1} u_{ym} e^{\lambda_m t} \sin k_m \xi \tag{S11}$$

with a positive integer m and eigenvalue λ_m of the time evolution operator, where k_m and ξ are defined as

$$k_m = m\pi \tag{S12}$$
$$\xi = \frac{y}{l}, \tag{S13}$$

respectively. Substituting equation (S11) into equation (S10), multiplying by $2\sin k_n \xi$ with a positive integer n, and integrating for $0 < y < l$, we obtain

$$\left\{\rho \lambda_m^2 + \eta_t \frac{k_m^2}{l^2}\lambda_m + E_1 \frac{k_m^2}{l^2} - \frac{(\mu_S - \mu_K) P_{ext}}{v_c \alpha_A H}\lambda_m\right\} u_n e^{\lambda_m t} = 0. \tag{S14}$$

From equations (S3) and (S14), we obtain

$$\rho L^2 \lambda_m^2 + k_m^2 \eta_t \left(\frac{A}{A_0}\right)^{-2} \lambda_m + k_m^2 E_1 \left(\frac{A}{A_0}\right)^{-2} - \frac{(\mu_S - \mu_K) P_{ext} L^2}{v_c \alpha_A H}\lambda_m = 0. \tag{S15}$$

The second term on the left-hand side of equation (S15) corresponds to the viscosity, while the fourth term on the same side represents the velocity-weakening friction. For $A/A_0 \ll 1$, we can neglect the fourth term and λ_m satisfies $\mathrm{Re}\,\lambda_m < 0$. For a larger A/A_0, the second and third terms can be neglected, and one of the solutions satisfies $\mathrm{Im}\,\lambda_m = 0$ and $\mathrm{Re}\,\lambda_m > 0$.

The fluctuation $\delta u_y(y,t)$ becomes unstable when $\mathrm{Re}\,\lambda_m > 0$; however, the oscillatory instability with $\mathrm{Im}\,\lambda_m \neq 0$ is suppressed by the static friction force because the oscillatory motion is accompanied by backward motion. Bulk sliding occurs when the eigenvalue satisfies

$$\mathrm{Im}\,\lambda_m = 0 \;\text{ and }\; \mathrm{Re}\,\lambda_m > 0 \tag{S16}$$

with $m = 1$. We obtain equation (4) in the main text for the critical area A_c from this condition using equation (S15), which gives

$$\frac{A_\mathrm{c}}{A_0} = \frac{\pi \eta_\mathrm{t}}{L\left(-\sqrt{\rho E_1} + \sqrt{\rho E_1 + \dfrac{(\mu_\mathrm{S} - \mu_\mathrm{K}) P_\mathrm{ext} \eta_\mathrm{t}}{v_\mathrm{c} \alpha_\mathrm{A} H}}\right)}. \tag{S17}$$

Macroscopic friction coefficient

The loading force F_N is given by

$$F_\mathrm{N} = P_\mathrm{ext} A_0. \tag{S18}$$

The driving force F_T is balanced with the total friction force, except for the duration of bulk sliding. It should be noted that the precursor motion is quasi-static. F_T is given by the integral of the local friction force as

$$F_\mathrm{T} = W \int_0^L \mu P_\mathrm{ext} dy. \tag{S19}$$

Using equations (2), (3), (S1), (S3), (S2), (S5), and (S6), we obtain

$$\begin{aligned} F_\mathrm{T} &= \alpha_\mathrm{A} H W \int_0^L \frac{d\sigma_{yy}(y)}{dy} dy \\ &= E_1 \alpha_\mathrm{A} H W \left\{ \int_0^l \frac{d^2 u_{y1}}{dy^2} dy + \int_l^L \frac{d^2 u_{y0}}{dy^2} dy \right\} \\ &= -E_1 \alpha_\mathrm{A} H W \left.\frac{du_{y1}}{dy}\right|_{y=0} \\ &= P_\mathrm{ext} A_0 \left\{ \mu_\mathrm{K} + (\mu_\mathrm{S} - \mu_\mathrm{K}) \frac{A}{A_0} \right\}. \end{aligned} \tag{S20}$$

Because the macroscopic static friction coefficient μ_M is the ratio of the driving force to the loading force, $F_\mathrm{T}/F_\mathrm{N}$, at $A = A_\mathrm{c}$, equation (6) in the main text is obtained from equations (S18) and (S20).

2.2. Model for large W/H

Quasi-static solution

The quasi-static solution $u_{y0}(x)$ of equations (9) and (10) in the main text at $U = 0$ is obtained as

$$u_{y0}(x) = \frac{\mu_\mathrm{K} P_\mathrm{ext}}{2 E_2 \alpha_\mathrm{B} H} \left(x^2 - W x\right) \tag{S21}$$

with the boundary conditions, local friction coefficient $\mu = \mu_{\mathrm{K}}$, and acceleration $\ddot{u}_y(x,t) = 0$. For $U > 0$, the quasi-static solution $u_y^{(\mathrm{a})}(x)$ is given by

$$u_y^{(\mathrm{a})}(x) = \begin{cases} u_{y1}(x), & 0 \le x \le \frac{w}{2} \\ u_{y0}(x), & \frac{w}{2} < x \le \frac{W}{2} \end{cases} \tag{S22}$$

with the width w of the precursor slip related to A as

$$A = wL. \tag{S23}$$

Setting μ equal to μ_{S} for $0 \le x \le w/2$, the displacement and stress are continuous at $x = w/2$:

$$u_{y1}\left(x = \tfrac{w}{2}\right) = u_{y0}\left(x = \tfrac{w}{2}\right) \tag{S24}$$

$$\left.\frac{du_{y1}}{dx}\right|_{x=\frac{w}{2}} = \left.\frac{du_{y0}}{dx}\right|_{x=\frac{w}{2}}, \tag{S25}$$

where we obtain $u_{y1}(x)$ as

$$u_{y1}(x) = u_{y0}(x) + \frac{(\mu_{\mathrm{S}} - \mu_{\mathrm{K}})P_{\mathrm{ext}}}{2E_2\alpha_{\mathrm{B}}H}\left(x^2 - wx + \frac{w^2}{4}\right) \tag{S26}$$

with

$$w = 2\left\{\frac{2E_2\alpha_{\mathrm{B}}HU}{(\mu_{\mathrm{S}} - \mu_{\mathrm{K}})P_{\mathrm{ext}}}\right\}^{\frac{1}{2}}. \tag{S27}$$

From equation (S23), A can be expressed as

$$\frac{A}{L} = 2\left\{\frac{2E_2\alpha_{\mathrm{B}}HU}{(\mu_{\mathrm{S}} - \mu_{\mathrm{K}})P_{\mathrm{ext}}}\right\}^{\frac{1}{2}}. \tag{S28}$$

Stability analysis

We introduce the fluctuation $\delta u_y(x,t)$ as

$$\delta u_y(x,t) = u_y(x,t) - u_y^{(\mathrm{a})}(x). \tag{S29}$$

Substituting equation (S29) into equation (9), we obtain

$$\rho\delta\ddot{u}_y = E_2\frac{\partial^2\delta u_y}{\partial x^2} + \frac{\eta_1}{2}\frac{\partial^2\delta\dot{u}_y}{\partial x^2} + \frac{(\mu_{\mathrm{S}} - \mu_{\mathrm{K}})P_{\mathrm{ext}}\delta\dot{u}_y}{v_{\mathrm{c}}\alpha_{\mathrm{B}}H}. \tag{S30}$$

Assuming $\delta u_y(x,t) = 0$ for $x > w/2$ and $x = 0$, $\delta u_y(x,t)$ is expressed as

$$\delta u_y(x,t) = \sum_{m=1} u_{ym}e^{\lambda_m t}\sin k_m\xi, \tag{S31}$$

where k_m and ξ are given by

$$k_m = m\pi \tag{S32}$$

$$\xi = \frac{2x}{w}. \tag{S33}$$

Here, m is a positive integer and λ_m is the eigenvalue. Substituting equation (S31) into equation (S30), multiplying by $2\sin k_n\xi$, and integrating for $0 < x < w/2$, we obtain

$$\left\{\rho\lambda_m^2 + 2\eta_1\frac{k_m^2}{w^2}\lambda_m + 4E_2\frac{k_m^2}{w^2} - \frac{(\mu_S-\mu_K)P_{ext}}{v_c\alpha_B H}\lambda_m\right\}u_n e^{\lambda_m t} = 0. \tag{S34}$$

From equations (S23) and (S34), we obtain

$$\rho W^2\lambda_m^2 + 2k_m^2\eta_1\left(\frac{A}{A_0}\right)^{-2}\lambda_m + 4k_m^2E_2\left(\frac{A}{A_0}\right)^{-2} - \frac{(\mu_S-\mu_K)P_{ext}W^2}{v_c\alpha_B H}\lambda_m = 0. \tag{S35}$$

The second term on the left-hand side of equation (S35) represents the viscosity, while the fourth term on the same side originates from the velocity-weakening friction.

Similar to the case for $L/H \gg 1$, bulk sliding occurs when the eigenvalue satisfies equation (S16) with increasing A/A_0 owing to the instability caused by the velocity-weakening friction. From this condition, we obtain equation (11) in the main text for the critical area A_c, which gives

$$\frac{A_c}{A_0} = \frac{\pi\eta_1}{W\left(-\sqrt{\rho E_2} + \sqrt{\rho E_2 + \frac{(\mu_S-\mu_K)P_{ext}\eta_1}{2v_c\alpha_B H}}\right)}. \tag{S36}$$

Macroscopic friction coefficient

The loading force F_N is given by equation (S18). The driving force F_T is balanced with the integral of the local friction force as

$$F_T = L\int_{-\frac{W}{2}}^{\frac{W}{2}}\mu P_{ext}dx = 2L\int_0^{\frac{W}{2}}\mu P_{ext}dx. \tag{S37}$$

Using equations (9), (10), (S21), (S23), (S22), (S25), and (S26), we obtain

$$\begin{aligned} F_T &= 2\alpha_B HL\int_0^{\frac{W}{2}}\frac{d\sigma_{xy}(x)}{dx}dx \\ &= 2E_2\alpha_B HL\left\{\int_0^{\frac{w}{2}}\frac{d^2u_{y1}}{dx^2}dx + \int_{\frac{w}{2}}^{\frac{W}{2}}\frac{d^2u_{y0}}{dx^2}dx\right\} \\ &= -2E_2\alpha_B HL\left.\frac{du_{y1}}{dx}\right|_{x=0} \\ &= P_{ext}A_0\left\{\mu_K + (\mu_S-\mu_K)\frac{A}{A_0}\right\}. \end{aligned} \tag{S38}$$

Because the macroscopic static friction coefficient μ_M is the ratio of the driving force to the loading force, F_T/F_N, at $A = A_c$, we obtain equation (6) in the main text from equations (S18) and (S38).

3. Comparison with the analytical result from a previous study

In this section, we compare our results with those of ref. 1. A previous study discussed the dependence of the precursor slip and friction coefficient on system size with a constant aspect

ratio $L/H = 2$. In the analysis, the authors considered the nonuniformity of the bottom pressure as the origin of the precursor slip and demonstrated the breakdown of Amontons' law. The bottom pressure was shown to increase along the driving direction owing to the torque effect[1,2] and was approximated by $p(y) = 2P_{\rm ext}y/L$. The equation of motion is given by

$$\rho \ddot{u}_y(y,t) = \frac{\partial \sigma_{yy}(y,t)}{\partial y} + \frac{\sigma_{zy}(y,t) - \mu(\dot{u}_y)p(y)}{\alpha H}, \tag{S39}$$

where the stresses σ_{yy} and σ_{zy} are expressed as

$$\sigma_{yy} = E_1 \frac{\partial u_y}{\partial y} + \eta_{\rm t} \frac{\partial \dot{u}_y}{\partial y} \tag{S40}$$

$$\sigma_{zy} = E_2 \frac{U - u_y}{H/2} + \frac{\eta_1}{2} \frac{V_{\rm rod} - \dot{u}_y}{H/2} \tag{S41}$$

with the fitting parameter α. The boundary conditions are as

$$\left.\frac{\partial u_y}{\partial y}\right|_{y=0} = \left.\frac{\partial u_y}{\partial y}\right|_{y=L} = 0. \tag{S42}$$

Using the same procedure as that in Sec. 2, the equation for the critical area $A_{\rm c}$ is obtained as

$$\frac{\pi^2 \eta_{\rm t}}{4}\left(\frac{A_{\rm c}}{A_0}\right)^{-2} + \frac{\eta_1}{\alpha}\left(\frac{L}{H}\right)^2 + 2L\sqrt{\rho\left\{\frac{\pi^2 E_1}{4}\left(\frac{A_{\rm c}}{A_0}\right)^{-2} + \frac{2E_2}{\alpha}\left(\frac{L}{H}\right)^2\right\}}$$
$$= \frac{(\pi^2-4)}{\pi^2}\frac{(\mu_{\rm S}-\mu_{\rm K})P_{\rm ext}L^2}{\alpha v_{\rm c} H}\frac{A_{\rm c}}{A_0}. \tag{S43}$$

For $A_{\rm c}/A_0 \ll 1$, $A_{\rm c}/A_0$ is expressed as

$$\frac{A_{\rm c}}{A_0} \simeq \frac{\pi^2}{\pi^2-4}\left(\frac{\mu_{\rm S}-\mu_{\rm K}}{\alpha}\right)^{-\frac{1}{3}}\left(\frac{P_{\rm ext}H}{\eta_{\rm t}v_{\rm c}}\right)^{-\frac{1}{3}}\left(\frac{L}{H}\right)^{-\frac{2}{3}}, \tag{S44}$$

where the exponents for $P_{\rm ext}$ and L/H differ from those in equation (5) in the main text. The difference in the exponents results from the different assumptions for the bottom pressure adopted in both studies, i.e., uniform pressure in the present study and non-uniform pressure in the previous study. Note that the macroscopic friction coefficient $\mu_{\rm M}$ in this model is also given by equation (6) in the main text.

In Fig. S3, we show $\mu_{\rm M}$ against $P_{\rm ext}$ for various lengths, L/H with $W/H = 1$, obtained from the FEM simulations in the present study. The analytical results in ref. 1 given by equations (S43) and (6), and in the present study given by equations (S17) and (6) are also shown. The fitting parameter α is determined such that the analytical result matches the FEM results for $L/H = 2$ and $P_{\rm ext}/E > 0.01$. Figure S3 shows that the analytical results in the previous study better reproduce the results of the FEM analysis for $L/H \leq 2$. This is because the nonuniformity of the bottom pressure is significant for $L/H \leq 2$. However, the analytical results deviate from the FEM analysis for $L/H \geq 4$, where the bottom pressure is almost uniform, as shown in Fig. S1 (c) and Supplementary Video S1 (c). Instead, the analytical results of the present study agree with the FEM analysis for $L/H \geq 4$, as shown in Fig. 3 and Fig. S3.

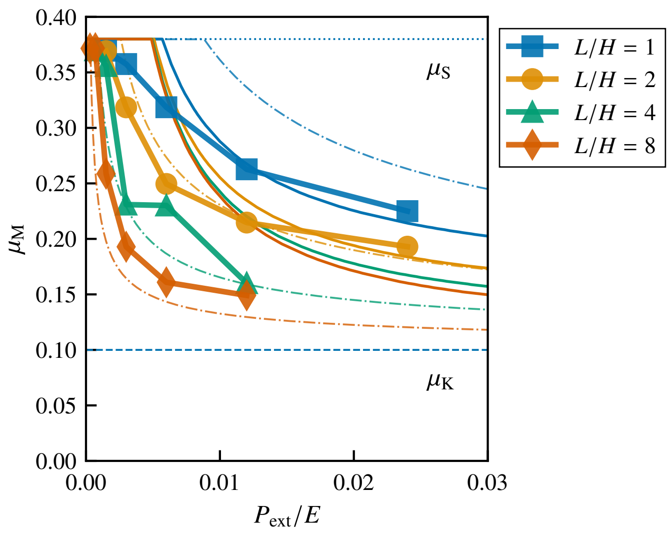

Figure S3. Dependence of μ_M on P_{ext} for various L/H values with $W/H = 1$. The thin solid lines represent the analytical results in ref. 1 given by equations (S43) and (6) with the fitting parameter $\alpha = 0.05$. The thin dash-dotted lines represent the analytical results in the present study given by equations (S17) and (6) with the fitting parameter $\alpha_A = 0.2$. The dotted and dashed lines represent μ_S and μ_K, respectively.

References

1. Otsuki, M. & Matsukawa, H. Systematic breakdown of Amontons' law of friction for an elastic object locally obeying Amontons' law. *Sci. Reports* **3**, 1586, DOI: https://doi.org/10.1038/srep01586 (2013).

2. Scheibert, J. & Dysthe, D. K. Role of friction-induced torque in stick-slip motion. *Europhys. Lett.* **92**, 54001, DOI: https://doi.org/10.1209/0295-5075/92/54001 (2010).
